# Our Prison Systems

**Published:** 1891-03

**Authors:** 


### HALL'S JOURNAL OF HEALTH

TRUTH DEMANDS NO SACRIFICE; ERROR CAN MAKE NONE.

Vol. 38. MARCH, 1891. No. 3.

### OUR PRISON SYSTEMS.

Much as our method of treating prisoners before and after conviction, has improved within the past few years, there is still abundant room for further improvement, especially in the jails and strongholds of our larger municipalities. It is a humane maxim of the law that an accused person is presumed to be innocent until he is proven guilty, but from the treatment many prisoners receive at the hands of petty officials whilst held to await the issue of a trial, the reverse proposition would seem to be the rule. Especially is this the case in the City of New York, where the jail life of accused persons is far from what it should be. It is hardly to be expected that humane gentle natured men would seek or retain the place of jail keepers, but surely there is no excuse for the exercise of undue severity in dealing with those unfortunates who, by the force of circumstances are temporarily committed to their charge. Much less is there excuse or palliation for the wretchedly fifthy and unhealthy state of many of the lock-ups and cells assigned to persons restrained of their liberty.

We have in mind the recent commitment of two refined and educated gentlemen (father and son) to the Tombs, upon a charge absolutely false, as it subsequently appeared, where they were not only subjected to the insolence and brutality of coarse subordinates, but the cell where they were compelled to spend their nights, was alive with vermin. A still more recent case of the same kind with like experiences, occurred in a neighboring city. It is true, that a little *salve to itching palms* might have softened the rigors of treatment meted out to those who decline to purchase forbearance at the expense of principle. Even criminals have rights which all men should respect, and among the more essential ones are the right to pure air which costs nothing and sanitary quarters and surroundings, which ought to be insisted on by those in authority at whatever cost.

What we have said of our jails or temporary prisons, applies with equal force to our more extensive prisons and reformatories, designed for malefactors after conviction. We as a people, have no right to add punishment to punishment by subjecting criminals to needless hardship, much less to shorten their lives by unhealthy conditions. There is to-day, in our prison houses, many an involuntary inmate, who, in all the qualities of manhood is greatly the superior of his keeper. Under some dire provocation, which not to resent, would have shown him less of a man, he incurred the penalty of confinement at hard labor for a term of years. The state is entitled to his earnings, but in its turn, it owes him humane treatment, comfortable clothing, pure air, healthful food and a reasonably clean and restful bed in the solitary hours.

Mr. Havelock Ellis, a well known Englishman, who has given much attention to this subject, in a recent treatise, says that the ratio of criminals in most civilized communities, including the United States, is greatly on the increase, and Sidney Smith once declared the prisons of England to be schools for the education of criminals. Are not ours open to the same charge ?

Nothing so tends to harden and embitter a criminal, as a sense of undue humiliation and severity. His very soul recoils against it, till he feels that he has much to revenge, and this feeling is a frequent source of continual ill-doing.

In theory, our prisons are reformatory institutions. The leading objects are the security of the State against the repetition of crime, and the reformation of offenders. That the best means of attaining these objects have not as yet been arrived at, all must admit. The subject is one which cannot too seriously engage the attention of our lawmakers.

We have held for a long time, that a system of prison labor which carries to the credit of the laborer the avails of his handiwork, deducting therefrom a reasonable sum for his keeping, and paying over to him the surplus, if any, at the time of his discharge, would secure more and better work in the various trades, and leave the discharged convict not altogether empty handed, upon his entering anew upon his struggle for subsistence, outside the gates. This may appear to be impracticable, but we would like to see it tried.

